# Synthesis and crystal structure of bis­[*trans*-di­aqua­(1,4,8,11-tetra­aza­cyclo­tetra­decane-κ^4^ *N* ^1^,*N* ^4^,*N* ^8^,*N* ^11^)nickel(II)] *trans*-(1,4,8,11-tetra­aza­cyclo­tetra­decane-κ^4^ *N* ^1^,*N* ^4^,*N* ^8^,*N* ^11^)bis­[4,4′,4′′-(1,3,5-tri­methyl­benzene-2,4,6-tri­yl)tris­(hydrogen phenyl­phospho­nato-κ*O*)]nickel(II) deca­hydrate

**DOI:** 10.1107/S2056989022006624

**Published:** 2022-06-30

**Authors:** Liudmyla V. Tsymbal, Rodinel Ardeleanu, Sergiu Shova, Yaroslaw D. Lampeka

**Affiliations:** a L. V. Pisarzhevskii Institute of Physical Chemistry of the National Academy of, Sciences of Ukraine, Prospekt Nauki, 31, Kiev 03028, Ukraine; b "Petru Poni" Institute of Macromolecular Chemistry, Department of, Inorganic, Polymers, Aleea Grigore Ghica Voda 41A, RO-700487 Iasi, Romania

**Keywords:** crystal structure, cyclam, nickel, tri­phospho­nic acid

## Abstract

The components of the title compound, [Ni(C_10_H_24_N_4_)(H_2_O)_2_]_2_[Ni(C_10_H_24_N_4_)(C_27_H_24_O_9_P_3_)_2_]·10H_2_O are two centrosymmetric [Ni(C_10_H_24_N_4_)(H_2_O)_2_]^2+^ dications, a centrosymmetric [Ni(C_10_H_24_N_4_)(C_27_H_24_O_9_P_3_)_2_]^4−^ tetra-anion and five crystallographically unique water mol­ecules of crystallization. All of the nickel ions are coordinated by the four secondary N atoms of the macrocyclic cyclam ligands, which adopt the most energetically stable *trans*-III conformation, and the mutually *trans* O atoms of either water mol­ecules in the cations or the phospho­nate groups in the anion in a tetra­gonally distorted NiN_4_O_2_ octa­hedral coordination geometry. Strong O—H⋯O hydrogen bonds between the protonated and the non-protonated phospho­nate O atoms of neighboring anions result in the formation of layers oriented parallel to the *bc* plane, which are linked into a three-dimensional network by virtue of numerous N—H⋯O and O—H⋯O hydrogen bonds arising from the *sec*-NH groups of the macrocycles, phospho­nate O atoms and coordinated and non-coordinated water mol­ecules.

## Abstract 2

The centrosymmetric *trans*-NiN_4_O_2_ coordination polyhedra of the Ni^2+^ ions in the complex cations and anions of the title compound are tetra­gonally distorted octa­hedra. In the crystal, O—H⋯O hydrogen bonds between the phospho­nate groups of the anions result in the formation of layers oriented parallel to the *bc* plane, which are further arranged into a three-dimensional network due to hydrogen-bonding involving the macrocyclic di-aqua cations and water mol­ecules.

## 1. Chemical context

First-row transition-metal complexes of 14-membered cyclam-like tetra­aza macrocyles (cyclam = 1,4,8,11-tetra­aza­cyclo­tetra­decane; C_10_H_24_N_4_; *L*) are characterized by high thermodynamic stability and kinetic inertness (Yatsimirskii & Lampeka, 1985 ▸) and are popular metal-containing building units for the construction of MOFs (Lampeka & Tsymbal, 2004 ▸; Suh & Moon, 2007 ▸; Suh *et al.*, 2012 ▸; Stackhouse & Ma, 2018 ▸). These crystalline coordination polymers, in which oligo­carboxyl­ates are the most common bridging ligands (Rao *et al.*, 2004 ▸), possess permanent porosity and demonstrate many promising applications in different areas (MacGillivray & Lukehart, 2014 ▸; Kaskel, 2016 ▸).

The rigid trigonal aromatic linker 1,3,5-benzene­tri­carboxyl­ate, C_9_H_3_O_6_^3–^, is widely used for the assembly of MOFs based on aza­macrocyclic cations (Lampeka & Tsymbal, 2004 ▸). Its tris-monodentate coordination in the *trans*-axial coordination positions of the metal ions leads predominantly to the formation of two-dimensional coordination polymers with hexa­gonal nets of 6^3^ topology (Alexandrov *et al.*, 2017 ▸). Usually, the modification of this bridge through the substitution of the carb­oxy­lic groups by *para*-carb­oxy­benzyl fragments (the ligand H_3_BTB, 4,4′,4′′-benzene-1,3,5-triyltri­benzoic acid) does not affect the coordination properties of the carboxyl­ate groups or the topological characteristics of polymeric nets but results in the enlargement of the hexa­gonal structural unit of the coordination polymers allowing inter­penetration of the subnets (Lampeka *et al.*, 2012 ▸; Gong *et al.*, 2016 ▸). Compared to carboxyl­ates, linkers with other coordinating functions, in particular oligo­phospho­nates, have been studied to a much lesser extent (Gagnon *et al.*, 2012 ▸; Firmino *et al.*, 2018 ▸; Yücesan *et al.*, 2018 ▸), though one can expect that the substitution of a mono-anionic carb­oxy­lic group by a di-anionic phospho­nate group with distinct acidity, number of donor atoms and spatial directivity of coordination bonds will strongly influence the composition and topology of the coordination nets. However, except for a very recent publication (Tsymbal *et al.*, 2022 ▸), no papers dealing with structural characterization of the complexes formed by metal aza­macrocyclic cations and phospho­nate ligands have been published to date. 
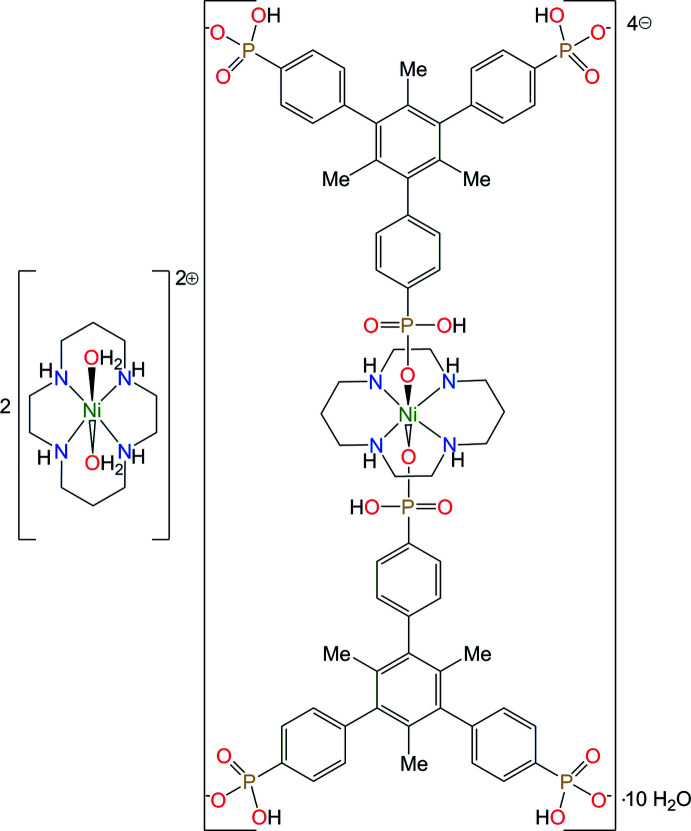


We report here the synthesis and crystal structure of the product of the reaction of [Ni(*L*)](ClO_4_)_2_ with 4,4′,4′′-(1,3,5-tri­methyl­benzene-2,4,6-tri­yl)tri­phospho­nic acid, H_6_Me_3_BTP) – the structural analogue of H_3_BTB, namely, bis­[*trans*-di­aqua-(1,4,8,11-tetra­aza­cyclo­tetra­decane-κ^4^ *N* ^1^,*N* ^4^,*N* ^8^,*N* ^11^)-nickel(II)] *trans*-{bis-[4,4′,4′′-(1,3,5-tri­methyl­benzene-2,4,6-tri­yl)tris­(hydrogen phenyl­phospho­nato-κ*O*)-(1,4,8,11-tetra­aza­cyclo­tetra­decane-κ^4^ *N* ^1^,*N* ^4^,*N* ^8^,*N* ^11^)-nickel(II)]} deca­hydrate, [Ni(*L*)(H_2_O)_2_]_2_[Ni(*L*)(H_3_Me_3_BTP)_2_]·10H_2_O, **I**.

## 2. Structural commentary

**Figure 1:**
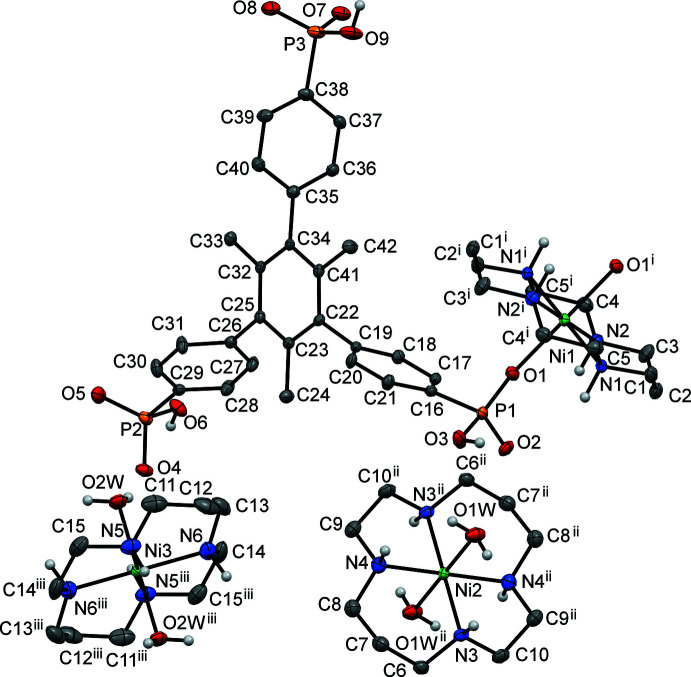
The extended asymmetric unit in **I** showing the coordination environment of the Ni atoms and the atom-labeling scheme (displacement ellipsoids are drawn at the 30% probability level). C-bound H atoms and uncoordinated water molecules are omitted for clarity. Symmetry codes: (i) −*x* + 2, −*y* + 1, −*z* + 2; (ii) −*x* + 2, −*y* + 2, −*z* + 1; (iii) −*x* + 1, −*y* + 3, −*z* + 1.

The mol­ecular structure of **I** is shown in Fig. 1 ▸. It represents a non-polymeric compound in which atom Ni1 is coordinated by two monodentate H_3_Me_3_BTP^3–^ ligands *via* their phospho­nate O atoms, resulting in the formation of an [Ni(*L*)(H_3_Me_3_BTP)_2_]^4–^ complex anion, which is charge-balanced by two structurally non-equivalent [Ni(*L*)(H_2_O)_2_]^2+^ divalent cations formed by atoms Ni2 and Ni3. The coordination geometries of all the nickel ions in **I** have much in common: the Ni^2+^ ions (all with site symmetry 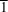 ) are coordinated by the four secondary N atoms of the macrocyclic ligands *L*, which adopt the most energetically stable *trans*-III (*R,R,S,S*) conformation (Bosnich *et al.*, 1965*a* ▸; Barefield *et al.*, 1986 ▸) with the five-membered (N—Ni—N bite angles ≃ 85°) and six-membered (N—Ni—N bite angles ≃ 95°) chelate rings in *gauche* and chair conformations, respectively (Table 1 ▸). The coordination polyhedra of the metal ions can be described as tetra­gonally elongated *trans*-NiN_4_O_2_ octa­hedra with the Ni—N bond lengths [average value 2.068 (3) Å] slightly shorter than the Ni—O bonds which, in turn, do not display any dependence on the nature of the donor oxygen atoms. The location of the metal ions on crystallographic inversion centers enforces strict planarity of the Ni(N_4_) coordination moieties and the axial Ni—O bonds are nearly orthogonal to the NiN_4_ planes (deviations of the angles N—Ni—O from 90° do not exceed 2°).

The pendant benzene rings of the H_3_Me_3_BTP^3–^ tri-anion in **I** are substanti­ally tilted relative to the central aromatic core [average angle between the mean planes = 76 (5)°] and this feature is caused by repulsive inter­actions between the hydrogen atoms of the pendant rings and those of the methyl substituents of the central ring. The P—OH bond lengths [average value 1.57 (3) Å] are larger than the other P—O bonds [average value 1.501 (5) Å], thus indicating the partially delocalized character of the phospho­nate groups.

## 3. Supra­molecular features

**Figure 2:**
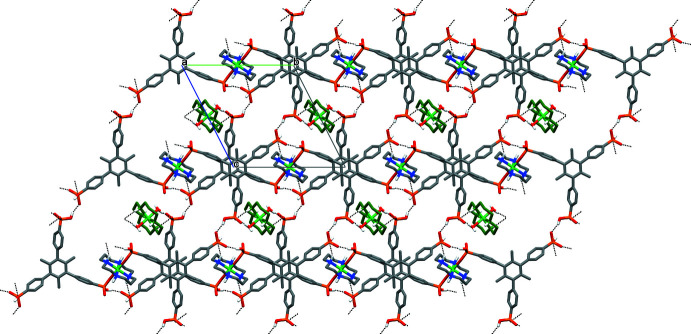
The hydrogen-bonded (dashed lines) layers in **I** viewed down the *a* axis. C-bound H atoms and macrocyclic cations formed by Ni3 have been omitted; C and N atoms of the macrocyclic cations formed by Ni2 are shown in green.

In the crystal of **I**, the [Ni1(*L*)(H_3_Me_3_BTP)_2_]^4–^ anions, [Ni2/Ni3(*L*)(H_2_O)_2_]^2+^ cations and water mol­ecules of crystallization are linked by numerous hydrogen bonds with participation of the phospho­nate groups, the secondary amino groups of the macrocycles and both the coordinated and crystalline water mol­ecules (Table 2 ▸). A distinct lamellar structure is inherent for this compound. In particular, strong hydrogen-bonding inter­actions between the protonated fragments of the P1 and P3 phospho­nate groups of one mol­ecule as the donors with the non-protonated O4 and O5 atoms of the P2 group of another mol­ecule as the acceptors [P1—O3—H3*C*⋯O5(*x*, *y* − 1, *z*), P3—O9—H9*C*⋯O49(*x*, *y* − 1, *z* + 1)], together with a weak N1—H1⋯O6 (*x*, *y* − 1, *z*) hydrogen bond between the secondary amino group of the macrocyclic cation [Ni1(*L*)] and protonated P2—O6 phospho­nate fragment result in the formation of anionic layers oriented parallel to the *bc* plane. The distance between the parallel mean planes of the staggered by 60° tri­methyl­benzene rings of neighboring H_3_Me_3_BTP^3–^ anions is 5.248 (3) Å, thus allowing us to exclude the possibility of aromatic π–π stacking inter­actions between them. Additionally, the negative charge of the layers are partially compensated by the incorporation within the layers of the [Ni2(*L*)(H_2_O)_2_]^2+^ cations *via* hydrogen bonding between the coordinated water mol­ecules and the phos­phon­ate O7 atom [O1*W*—H1W*B*⋯O7(*x*, *y*, z − 1)] (Fig. 2 ▸).

**Figure 3:**
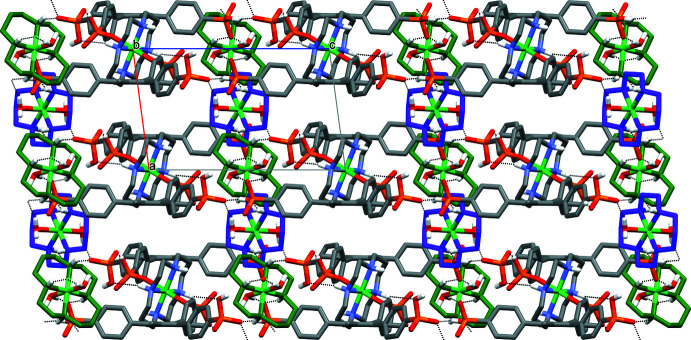
The structure of **I** viewed down the *b* axis. C-bound H atoms have been omitted; C and N atoms of the macrocyclic cation formed by Ni2 and Ni3 are shown in green and violet, respectively. Water mol­ecules of crystallization are not shown; hydrogen bonds are depicted as dashed lines.

The second macrocyclic aqua cation [Ni3(*L*)(H_2_O)_2_]^2+^, due to hydrogen bonding of the coordinated water mol­ecule with the phospho­nate O4 atom (O2*W*—H2*WB*⋯O4), serves as the bridge between the layers, arranging them into a three-dimensional network (Fig. 3 ▸), which is further stabilized by numerous O—H⋯O hydrogen bonds involving the water mol­ecules of crystallization, O3*W*–O7*W* (Table 2 ▸).

## 4. Database survey

A search of the Cambridge Structural Database (CSD, version 5.43, last update March 2022; Groom *et al.*, 2016 ▸) gave no hits related to H_6_Me_3_BTP or its complexes with metal ions, so the present work is the first structural characterization of a complex of this ligand. At the same time, several works dealing with the structures of the non-methyl­ated analogue of the phospho­nate under consideration, namely, 4,4′,4′′-benzene-1,3,5-triyl-tri­phospho­nic acid (H_6_BTP), have been published. They include a methanol solvate of the free acid (CSD refcode AKEPEO; Vilela *et al.*, 2021 ▸) and its pyridinium salt (YOLGEM; Beckmann *et al.*, 2008 ▸), mol­ecular complexes with solvated Co^II^ and Ni^II^ ions (OQIZAR and OQIZEV; Pili *et al.*, 2016 ▸) and coordination polymers formed by Sr^II^ (SOTZOR; Vaidhyanathan *et al.*, 2009 ▸), Zn^II^ (ISELAV02; Hermer *et al.*, 2016 ▸), Y^III^ (AKEPOY; Vilela *et al.*, 2021 ▸), Zr^IV^ (COCLIR; Taddei *et al.*, 2014 ▸) and V^IV/V^ (COQNAY; Ouellette *et al.*, 2009 ▸). Inter­estingly, as in **I**, in all the metal complexes except COCLIR and ISELAV02, the ligand acts as a H_3_BTP^3–^ tri-anion with three monodeprotonated phos­phon­ate groups. On the other hand, because of the absence of methyl substituents, the mol­ecules of the anions H_*n*_BTP^(6–*n*)–^ as a whole are flatter than H_3_Me_3_BTP^3–^ in **I** with a maximal tilting angle of pendant *versus* central benzene rings of *ca* 49° observed in ISELAV02. In addition, in the majority of complexes formed by H_*n*_BTP^(6–*n*)–^ ligands (except AKEPOY and ISELAV02), aromatic π–π stacking inter­actions of different strengths are observed with centroid-to-centroid distances between the central aromatic rings ranging from 3.4 to 3.9 Å.

The Cambridge Structural Database contains also 18 hits describing the structure of the [Ni(*L*)(H_2_O)_2_]^2+^ complex cation in salts of different inorganic and organic anions as well as the charge-compensating part in anionic coordination polymers. In general, the structure of this cation in **I** is similar to other compounds, both from the point of view of the conformation of the macrocycle and the bond distances and angles characterizing the coordination polyhedron of the metal.

## 5. Synthesis and crystallization

All chemicals and solvents used in this work were purchased from Sigma–Aldrich and used without further purification. The acid H_6_Me_3_BTP was synthesized according to a procedure described previously for the preparation of H_6_BTP (Vaidhyanathan *et al.*, 2009 ▸), starting from 1,3,5-trimethyl-2,4,6-tris­(4′-bromo­phen­yl)benzene instead of 1,3,5-tris­(4′-bromo­phen­yl)benzene. The complex [Ni(*L*)](ClO_4_)_2_ was prepared from ethanol solutions as described in the literature (Bosnich *et al.*, 1965*b* ▸).

The title compound **[Ni(** ***L*** **)(H_2_O)_2_]_2_[Ni(** ***L*** **)(H_3_Me_3_BTP)_2_]·10H_2_O**, **I**, was prepared as follows. A solution of [Ni(*L*)](ClO_4_)_2_ (46 mg, 0.1 mmol) in 5 ml of water was added to 5 ml of an aqueous solution of H_6_Me_3_BTP (18 mg, 0.03 mmol) containing 2 ml of pyridine. The pink precipitate, which formed in a week, was filtered off, washed with small amounts of water, methanol and diethyl ether, and dried in air. Yield: 7 mg (10% based on acid). Analysis calculated for C_84_H_148_N_12_Ni_3_O_32_P_6_: C 45.85, H 6.78, N 7.64%. Found: C 45.73, H 6.87, N 7.51%. Single crystals of **I** suitable for X-ray diffraction analysis were selected from the sample resulting from the synthesis.

**Caution! Perchlorate salts of metal complexes are potentially explosive and should be handled with care**.

## 6. Refinement

Crystal data, data collection and structure refinement details are summarized in Table 3 ▸. H atoms in **I** were placed in geometrically idealized positions and constrained to ride on their parent atoms, with C—H distances of 0.95 Å (ring H atoms), 0.98 Å (methyl H atoms), 0.99 Å (methyl­ene H atoms), N—H distances of 1.00 Å, O—H distances of 0.84 Å (protonated phospho­nate groups) and 0.87 Å (water mol­ecules) with *U*_iso_(H) values of 1.2*U*_eq_ or 1.5*U*_eq_ times those of the corresponding parent atoms.

## Supplementary Material

Crystal structure: contains datablock(s) I. DOI: 10.1107/S2056989022006624/hb8026sup1.cif

Structure factors: contains datablock(s) I. DOI: 10.1107/S2056989022006624/hb8026Isup2.hkl

CCDC reference: 2178456

Additional supporting information:  crystallographic information; 3D view; checkCIF report

## Figures and Tables

**Table 1:** Selected geometric parameters (Å, °)

Ni1—N1	2.067 (4)	Ni2—O1*W*	2.105 (4)
Ni1—N2	2.064 (4)	Ni3—N5	2.070 (4)
Ni1—O1	2.134 (3)	Ni3—N6	2.056 (5)
Ni2—N3	2.072 (4)	Ni3—O2*W*	2.136 (3)
Ni2—N4	2.076 (4)		
			
N1—Ni1—N2^i^	85.31 (16)	N3—Ni2—N4	95.34 (16)
N1—Ni1—N2	94.69 (16)	N5—Ni3—N6^iii^	85.2 (2)
N3—Ni2—N4^ii^	84.66 (16)	N5—Ni3—N6	94.8 (2)

Symmetry codes: (i) 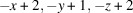 ; (ii) 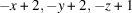 ; (iii) 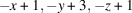 .

**Table 2:** Hydrogen-bond geometry (Å, °)

*D*—H⋯*A*	*D*—H	H⋯*A*	*D*⋯*A*	*D*—H⋯*A*
N1—H1⋯O6^iv^	1.00	2.32	3.196 (5)	146
N2—H2⋯O6*W*	1.00	2.18	3.039 (6)	143
N3—H3⋯O7^v^	1.00	2.13	3.102 (6)	162
N4—H4⋯O4*W*	1.00	2.06	3.056 (6)	173
N5—H5⋯O9^vi^	1.00	2.07	3.003 (6)	155
N6—H6⋯O7*W* ^ii^	1.00	1.98	2.956 (6)	166
O3—H3*C*⋯O5^iv^	0.84	1.84	2.654 (5)	162
O6—H6*C*⋯O3*W* ^vii^	0.84	1.75	2.550 (5)	159
O9—H9*C*⋯O4^viii^	0.84	1.74	2.517 (5)	154
O1*W*—H1*WB*⋯O7^v^	0.87	1.81	2.679 (5)	173
O1*W*—H1*WA*⋯O4*W*	0.87	2.45	3.256 (6)	155
O2*W*—H2*WB*⋯O4	0.86	1.90	2.729 (5)	164
O2*W*—H2*WA*⋯O7*W* ^ix^	0.86	1.81	2.675 (6)	174
O3*W*—H3*WB*⋯O2	0.87	1.81	2.676 (4)	177
O3*W*—H3*WA*⋯O7^v^	0.85	1.84	2.689 (5)	174
O4*W*—H4*WB*⋯O3	0.87	2.26	3.115 (6)	167
O4*W*—H4*WA*⋯O8^v^	0.87	1.93	2.796 (6)	172
O5*W*—H5*WB*⋯O5^x^	0.87	1.98	2.813 (5)	159
O5*W*—H5*WA*⋯O8^xi^	0.87	1.87	2.725 (5)	168
O6*W*—H6*WB*⋯O2	0.87	2.02	2.799 (6)	149
O6*W*—H6*WA*⋯O5*W*	0.87	2.00	2.842 (5)	164
O7*W*—H7*WB*⋯O3*W*	0.85	2.02	2.731 (5)	140
O7*W*—H7*WA*⋯O5*W*	0.86	1.83	2.688 (5)	173

Symmetry codes: (ii) 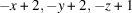 ; (iv) 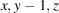 ; (v) 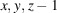 ; (vi) 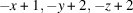 ; (vii) 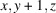 ; (viii) 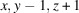 ; (ix) 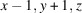 ; (x) 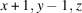 ; (xi) 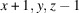 .

**Table 3:** Experimental details

Crystal data	
Chemical formula	[Ni(C_10_H_24_N_4_)(H_2_O)_2_]_2_[Ni(C_10_H_24_N_4_)(C_27_H_24_O_9_P_3_)_2_]·10H_2_O
*M*_r_	2200.09
Crystal system, space group	Triclinic, *P* 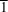
Temperature (K)	160
*a*, *b*, *c* (Å)	9.8779 (5), 17.2467 (11), 17.6707 (11)
α, β, γ (°)	61.409 (6), 77.515 (5), 77.713 (5)
*V* (Å^3^)	2559.7 (3)
*Z*	1
Radiation type	Mo *K*α
μ (mm^−1^)	0.72
Crystal size (mm)	0.40 × 0.10 × 0.10

Data collection	
Diffractometer	Rigaku Xcalibur Eos
Absorption correction	Multi-scan (*CrysAlis PRO*; Rigaku OD, 2020 ▸)
*T*_min_, *T*_max_	0.701, 1.000
No. of measured, independent and observed [*I* > 2σ(*I*)] reflections	23737, 9657, 6598
*R*_int_	0.063
(sin θ/λ)_max_ (Å^−1^)	0.610

Refinement	
*R*[*F* ^2^ > 2σ(*F* ^2^)], *wR*(*F* ^2^), *S*	0.067, 0.161, 1.02
No. of reflections	9657
No. of parameters	629
No. of restraints	1
H-atom treatment	H-atom parameters constrained
Δρ_max_, Δρ_min_ (e Å^−3^)	0.67, −0.46

Computer programs: *CrysAlis PRO* (Rigaku OD, 2020 ▸), *SHELXT* (Sheldrick, 2015*a* ▸), *SHELXL2018/3* (Sheldrick, 2015*b* ▸), *Mercury* (Macrae *et al.*, 2020 ▸) and *publCIF* (Westrip, 2010 ▸).

## supplementary crystallographic information

### Crystal data

**Table:**

[Ni(C_10_H_24_N_4_)(H_2_O)_2_]_2_[Ni(C_10_H_24_N_4_)(C_27_H_24_O_9_P_3_)_2_]·10H_2_O	*Z* = 1
*M**_r_* = 2200.09	*F*(000) = 1166
Triclinic, *P*1	*D*_x_ = 1.427 Mg m^−^^3^
*a* = 9.8779 (5) Å	Mo *K*α radiation, λ = 0.71073 Å
*b* = 17.2467 (11) Å	Cell parameters from 5403 reflections
*c* = 17.6707 (11) Å	θ = 2.1–26.3°
α = 61.409 (6)°	µ = 0.72 mm^−^^1^
β = 77.515 (5)°	*T* = 160 K
γ = 77.713 (5)°	Prism, clear light colourless
*V* = 2559.7 (3) Å^3^	0.40 × 0.10 × 0.10 mm

### Data collection

**Table:**

Rigaku Xcalibur Eos diffractometer	9657 independent reflections
Radiation source: fine-focus sealed X-ray tube, Enhance (Mo) X-ray Source	6598 reflections with *I* > 2σ(*I*)
Graphite monochromator	*R*_int_ = 0.063
Detector resolution: 16.1593 pixels mm^-1^	θ_max_ = 25.7°, θ_min_ = 2.1°
ω scans	*h* = −11→12
Absorption correction: multi-scan (CrysAlisPro; Rigaku OD, 2020)	*k* = −21→20
*T*_min_ = 0.701, *T*_max_ = 1.000	*l* = −21→21
23737 measured reflections	

### Refinement

**Table:**

Refinement on *F*^2^	Primary atom site location: structure-invariant direct methods
Least-squares matrix: full	Hydrogen site location: mixed
*R*[*F*^2^ > 2σ(*F*^2^)] = 0.067	H-atom parameters constrained
*wR*(*F*^2^) = 0.161	*w* = 1/[σ^2^(*F*_o_^2^) + (0.0527*P*)^2^ + 4.2781*P*] where *P* = (*F*_o_^2^ + 2*F*_c_^2^)/3
*S* = 1.02	(Δ/σ)_max_ < 0.001
9657 reflections	Δρ_max_ = 0.67 e Å^−^^3^
629 parameters	Δρ_min_ = −0.46 e Å^−^^3^
1 restraint	

### Special details

**Table:**

Geometry. All esds (except the esd in the dihedral angle between two l.s. planes) are estimated using the full covariance matrix. The cell esds are taken into account individually in the estimation of esds in distances, angles and torsion angles; correlations between esds in cell parameters are only used when they are defined by crystal symmetry. An approximate (isotropic) treatment of cell esds is used for estimating esds involving l.s. planes.

### Fractional atomic coordinates and isotropic or equivalent isotropic displacement parameters (Å^2^)

**Table:**

	*x*	*y*	*z*	*U*_iso_*/*U*_eq_	
Ni1	1.000000	0.500000	1.000000	0.0228 (2)	
P2	0.77348 (14)	1.51832 (8)	0.73137 (8)	0.0273 (3)	
P1	0.86291 (13)	0.70402 (8)	0.82874 (8)	0.0243 (3)	
P3	0.75955 (15)	0.78200 (9)	1.49060 (8)	0.0332 (3)	
O5	0.6838 (4)	1.5710 (2)	0.7750 (2)	0.0380 (9)	
C29	0.7561 (5)	1.4015 (3)	0.7965 (3)	0.0241 (10)	
O4	0.7456 (4)	1.5452 (2)	0.6413 (2)	0.0333 (8)	
O3W	1.0380 (3)	0.6623 (2)	0.6411 (2)	0.0311 (8)	
H3WA	0.998703	0.703601	0.598132	0.047*	
H3WB	1.016063	0.687031	0.675752	0.047*	
O4W	0.6258 (4)	0.8895 (3)	0.6511 (3)	0.0607 (12)	
H4WA	0.622702	0.869018	0.615193	0.091*	
H4WB	0.655562	0.843018	0.696393	0.091*	
C22	0.7292 (4)	0.9758 (3)	0.9717 (3)	0.0193 (10)	
C38	0.7501 (5)	0.8505 (3)	1.3753 (3)	0.0274 (11)	
C26	0.7310 (5)	1.2190 (3)	0.9041 (3)	0.0230 (10)	
N1	0.9914 (4)	0.4354 (3)	0.9286 (2)	0.0275 (9)	
H1	0.956271	0.481640	0.873064	0.033*	
O3	0.7242 (4)	0.7049 (2)	0.7985 (2)	0.0387 (9)	
H3C	0.724711	0.656089	0.798510	0.058*	
C34	0.7272 (4)	0.9926 (3)	1.1008 (3)	0.0211 (10)	
N2	1.1919 (4)	0.5419 (3)	0.9365 (3)	0.0330 (10)	
H2	1.173656	0.596679	0.881233	0.040*	
C3	1.2935 (5)	0.4794 (4)	0.9119 (4)	0.0421 (14)	
H3A	1.320107	0.425875	0.965248	0.051*	
H3B	1.378830	0.507709	0.878884	0.051*	
O2	0.9773 (4)	0.7334 (2)	0.7517 (2)	0.0349 (8)	
O9	0.7490 (5)	0.6880 (2)	1.5017 (2)	0.0490 (11)	
H9C	0.757613	0.649777	1.553173	0.074*	
C35	0.7357 (5)	0.9486 (3)	1.1963 (3)	0.0245 (10)	
C41	0.7258 (5)	0.9377 (3)	1.0620 (3)	0.0229 (10)	
C23	0.7243 (5)	1.0683 (3)	0.9212 (3)	0.0229 (10)	
C30	0.6330 (5)	1.3737 (3)	0.8509 (3)	0.0335 (12)	
H30	0.555907	1.416974	0.852158	0.040*	
C25	0.7243 (5)	1.1221 (3)	0.9602 (3)	0.0216 (10)	
C5	0.8806 (6)	0.3769 (4)	0.9791 (3)	0.0381 (13)	
H5A	0.916444	0.324112	1.029994	0.046*	
H5B	0.851180	0.356262	0.942372	0.046*	
C33	0.7319 (5)	1.1422 (3)	1.0923 (3)	0.0280 (11)	
H33A	0.769682	1.105369	1.147602	0.042*	
H33B	0.637231	1.170079	1.102996	0.042*	
H33C	0.791865	1.188533	1.053584	0.042*	
C39	0.6285 (5)	0.9036 (3)	1.3453 (3)	0.0321 (12)	
H39	0.549541	0.906986	1.385777	0.039*	
C42	0.7241 (6)	0.8387 (3)	1.1158 (3)	0.0333 (12)	
H42A	0.818887	0.807974	1.111379	0.050*	
H42B	0.661713	0.818665	1.094399	0.050*	
H42C	0.690678	0.825159	1.176739	0.050*	
O7	0.9019 (4)	0.7838 (2)	1.5071 (2)	0.0399 (9)	
C36	0.8576 (5)	0.8993 (3)	1.2269 (3)	0.0283 (11)	
H36	0.938419	0.898840	1.186477	0.034*	
C19	0.7533 (4)	0.9154 (3)	0.9299 (3)	0.0194 (10)	
C21	0.6772 (5)	0.8176 (3)	0.8889 (3)	0.0282 (11)	
H21	0.603252	0.793241	0.884480	0.034*	
O8	0.6378 (4)	0.8126 (3)	1.5414 (2)	0.0476 (10)	
C20	0.6477 (5)	0.8787 (3)	0.9222 (3)	0.0287 (11)	
H20	0.553187	0.895677	0.940187	0.034*	
C2	1.2358 (6)	0.4512 (4)	0.8569 (3)	0.0407 (14)	
H2A	1.196217	0.505379	0.808660	0.049*	
H2B	1.314402	0.421415	0.830904	0.049*	
C37	0.8665 (5)	0.8500 (3)	1.3151 (3)	0.0306 (12)	
H37	0.952338	0.815768	1.334400	0.037*	
C32	0.7271 (4)	1.0848 (3)	1.0504 (3)	0.0211 (10)	
C24	0.7140 (6)	1.1079 (3)	0.8254 (3)	0.0314 (12)	
H24A	0.660985	1.072081	0.815897	0.047*	
H24B	0.808141	1.108441	0.793050	0.047*	
H24C	0.666278	1.168989	0.805139	0.047*	
Ni2	1.000000	1.000000	0.500000	0.0233 (2)	
O1W	0.9604 (4)	0.8687 (2)	0.5872 (2)	0.0452 (10)	
H1WA	0.881040	0.872384	0.618973	0.068*	
H1WB	0.944450	0.844264	0.557414	0.068*	
N3	0.9605 (4)	0.9803 (3)	0.4004 (2)	0.0297 (10)	
H3	0.924717	0.921388	0.427638	0.036*	
C10	1.0986 (5)	0.9720 (4)	0.3496 (3)	0.0380 (13)	
H10A	1.128892	1.031639	0.311897	0.046*	
H10B	1.091906	0.945314	0.312116	0.046*	
N4	0.7940 (5)	1.0447 (3)	0.5321 (3)	0.0377 (11)	
H4	0.744740	0.990779	0.568716	0.045*	
C9	0.7973 (6)	1.0856 (4)	0.5889 (3)	0.0408 (14)	
H9A	0.703665	1.090138	0.621780	0.049*	
H9B	0.824723	1.146279	0.553175	0.049*	
C7	0.7183 (6)	1.0577 (4)	0.4022 (3)	0.0418 (14)	
H7A	0.689758	0.997677	0.440380	0.050*	
H7B	0.646435	1.092349	0.362786	0.050*	
C6	0.8543 (5)	1.0479 (3)	0.3477 (3)	0.0350 (12)	
H6A	0.837243	1.030970	0.304733	0.042*	
H6B	0.891090	1.106025	0.315290	0.042*	
C8	0.7168 (6)	1.1019 (4)	0.4586 (4)	0.0434 (14)	
H8A	0.758644	1.158069	0.423021	0.052*	
H8B	0.618830	1.116899	0.480465	0.052*	
Ni3	0.500000	1.500000	0.500000	0.0280 (2)	
O2W	0.5104 (4)	1.5055 (2)	0.6168 (2)	0.0374 (9)	
H2WA	0.451405	1.548647	0.620771	0.056*	
H2WB	0.589347	1.520291	0.613928	0.056*	
N5	0.3476 (5)	1.4161 (3)	0.5683 (3)	0.0436 (12)	
H5	0.333782	1.392139	0.529340	0.052*	
O7W	1.3235 (4)	0.6425 (3)	0.6174 (3)	0.0660 (14)	
H7WA	1.366682	0.665639	0.637536	0.099*	
H7WB	1.244232	0.671069	0.624896	0.099*	
N6	0.6644 (5)	1.3992 (3)	0.5280 (3)	0.0464 (12)	
H6	0.671366	1.374659	0.486100	0.056*	
C14	0.7918 (6)	1.4389 (5)	0.5068 (4)	0.0558 (18)	
H14A	0.873557	1.397569	0.499126	0.067*	
H14B	0.802863	1.450247	0.554899	0.067*	
O6W	1.2369 (4)	0.7283 (3)	0.7946 (2)	0.0541 (11)	
H6WA	1.317921	0.726218	0.763661	0.081*	
H6WB	1.177461	0.729258	0.764271	0.081*	
O5W	1.4763 (3)	0.7022 (2)	0.6828 (2)	0.0395 (9)	
H5WA	1.538341	0.732237	0.641517	0.059*	
H5WB	1.524801	0.660758	0.722817	0.059*	
O1	0.9002 (4)	0.6178 (2)	0.9039 (2)	0.0326 (8)	
O6	0.9335 (4)	1.5164 (2)	0.7321 (2)	0.0402 (9)	
H6C	0.960853	1.564111	0.692096	0.060*	
C17	0.9212 (5)	0.8304 (3)	0.8664 (3)	0.0229 (10)	
H17	1.015334	0.815323	0.846193	0.027*	
C16	0.8155 (5)	0.7918 (3)	0.8618 (3)	0.0216 (10)	
C18	0.8904 (5)	0.8913 (3)	0.9005 (3)	0.0238 (10)	
H18	0.964113	0.916614	0.903815	0.029*	
C40	0.6201 (5)	0.9525 (3)	1.2561 (3)	0.0302 (12)	
H40	0.535465	0.988328	1.236326	0.036*	
C1	1.1247 (6)	0.3895 (3)	0.9048 (3)	0.0375 (13)	
H1A	1.108999	0.364727	0.867554	0.045*	
H1B	1.157958	0.339182	0.958205	0.045*	
C28	0.8659 (5)	1.3371 (3)	0.7957 (3)	0.0323 (12)	
H28	0.950712	1.353974	0.758385	0.039*	
C31	0.6206 (5)	1.2844 (3)	0.9032 (3)	0.0319 (12)	
H31	0.534822	1.267424	0.939145	0.038*	
C13	0.6486 (8)	1.3227 (4)	0.6163 (4)	0.0614 (19)	
H13A	0.651043	1.342483	0.660143	0.074*	
H13B	0.728211	1.275571	0.620745	0.074*	
C15	0.2154 (6)	1.4748 (5)	0.5758 (4)	0.0502 (16)	
H15A	0.212231	1.487228	0.625394	0.060*	
H15B	0.133940	1.444182	0.586810	0.060*	
C11	0.3777 (8)	1.3401 (5)	0.6484 (4)	0.0606 (19)	
H11A	0.300674	1.302890	0.671624	0.073*	
H11B	0.383358	1.360212	0.691424	0.073*	
C27	0.8528 (5)	1.2485 (3)	0.8489 (3)	0.0315 (12)	
H27	0.930342	1.205552	0.847868	0.038*	
C12	0.5140 (8)	1.2849 (4)	0.6361 (4)	0.065 (2)	
H12A	0.519087	1.227524	0.689642	0.078*	
H12B	0.509541	1.271311	0.588269	0.078*	
C4	1.2423 (5)	0.5705 (4)	0.9910 (3)	0.0360 (13)	
H4A	1.318132	0.607996	0.956861	0.043*	
H4B	1.279137	0.517994	1.041693	0.043*	

### Atomic displacement parameters (Å^2^)

**Table:**

	*U*^11^	*U*^22^	*U*^33^	*U*^12^	*U*^13^	*U*^23^
Ni1	0.0278 (5)	0.0183 (4)	0.0225 (4)	0.0031 (4)	−0.0059 (3)	−0.0108 (4)
P2	0.0380 (8)	0.0178 (6)	0.0273 (7)	−0.0020 (6)	−0.0117 (6)	−0.0089 (5)
P1	0.0314 (7)	0.0195 (6)	0.0258 (6)	0.0026 (6)	−0.0079 (5)	−0.0139 (5)
P3	0.0495 (9)	0.0290 (7)	0.0222 (7)	−0.0109 (7)	−0.0112 (6)	−0.0074 (6)
O5	0.056 (2)	0.0220 (18)	0.042 (2)	0.0005 (18)	−0.0129 (18)	−0.0184 (17)
C29	0.031 (3)	0.023 (2)	0.019 (2)	0.000 (2)	−0.0063 (19)	−0.010 (2)
O4	0.051 (2)	0.0229 (18)	0.0265 (18)	−0.0077 (17)	−0.0132 (16)	−0.0072 (15)
O3W	0.0317 (19)	0.0298 (19)	0.0354 (19)	0.0021 (16)	−0.0082 (15)	−0.0185 (16)
O4W	0.064 (3)	0.059 (3)	0.060 (3)	−0.013 (2)	−0.008 (2)	−0.026 (2)
C22	0.017 (2)	0.022 (2)	0.020 (2)	−0.002 (2)	0.0003 (18)	−0.012 (2)
C38	0.040 (3)	0.022 (2)	0.025 (3)	−0.007 (2)	−0.011 (2)	−0.010 (2)
C26	0.030 (3)	0.019 (2)	0.022 (2)	−0.002 (2)	−0.005 (2)	−0.011 (2)
N1	0.036 (2)	0.021 (2)	0.026 (2)	0.0064 (19)	−0.0090 (18)	−0.0131 (18)
O3	0.049 (2)	0.027 (2)	0.054 (2)	0.0082 (18)	−0.0240 (18)	−0.0272 (19)
C34	0.019 (2)	0.025 (2)	0.021 (2)	0.006 (2)	−0.0042 (18)	−0.014 (2)
N2	0.039 (3)	0.031 (2)	0.030 (2)	−0.003 (2)	−0.0034 (19)	−0.016 (2)
C3	0.033 (3)	0.052 (4)	0.046 (3)	−0.004 (3)	0.006 (2)	−0.032 (3)
O2	0.047 (2)	0.031 (2)	0.0312 (19)	−0.0057 (17)	0.0045 (16)	−0.0216 (17)
O9	0.090 (3)	0.034 (2)	0.028 (2)	−0.021 (2)	−0.022 (2)	−0.0065 (17)
C35	0.028 (3)	0.023 (2)	0.024 (2)	−0.004 (2)	−0.004 (2)	−0.012 (2)
C41	0.023 (2)	0.019 (2)	0.027 (2)	0.000 (2)	−0.0026 (19)	−0.012 (2)
C23	0.026 (3)	0.022 (2)	0.019 (2)	0.004 (2)	−0.0033 (19)	−0.010 (2)
C30	0.028 (3)	0.022 (3)	0.046 (3)	0.003 (2)	−0.006 (2)	−0.014 (2)
C25	0.021 (2)	0.018 (2)	0.025 (2)	0.001 (2)	−0.0017 (19)	−0.010 (2)
C5	0.053 (4)	0.030 (3)	0.034 (3)	−0.007 (3)	−0.010 (3)	−0.014 (2)
C33	0.035 (3)	0.022 (2)	0.031 (3)	0.000 (2)	−0.008 (2)	−0.016 (2)
C39	0.033 (3)	0.040 (3)	0.023 (3)	−0.007 (3)	−0.003 (2)	−0.013 (2)
C42	0.052 (3)	0.021 (3)	0.027 (3)	−0.004 (2)	−0.009 (2)	−0.010 (2)
O7	0.053 (2)	0.038 (2)	0.0282 (19)	−0.0107 (19)	−0.0119 (17)	−0.0096 (17)
C36	0.038 (3)	0.025 (3)	0.024 (3)	0.001 (2)	−0.006 (2)	−0.014 (2)
C19	0.023 (2)	0.018 (2)	0.015 (2)	0.001 (2)	−0.0015 (18)	−0.0077 (19)
C21	0.026 (3)	0.032 (3)	0.036 (3)	−0.002 (2)	−0.006 (2)	−0.023 (2)
O8	0.054 (2)	0.053 (3)	0.0258 (19)	−0.001 (2)	−0.0044 (17)	−0.0116 (19)
C20	0.023 (3)	0.033 (3)	0.036 (3)	0.004 (2)	−0.001 (2)	−0.024 (2)
C2	0.036 (3)	0.048 (4)	0.041 (3)	0.010 (3)	−0.005 (2)	−0.029 (3)
C37	0.038 (3)	0.025 (3)	0.030 (3)	0.007 (2)	−0.015 (2)	−0.013 (2)
C32	0.020 (2)	0.023 (2)	0.022 (2)	0.004 (2)	−0.0025 (18)	−0.013 (2)
C24	0.050 (3)	0.021 (2)	0.025 (3)	−0.003 (2)	−0.007 (2)	−0.012 (2)
Ni2	0.0292 (5)	0.0196 (4)	0.0220 (4)	0.0031 (4)	−0.0055 (4)	−0.0117 (4)
O1W	0.073 (3)	0.036 (2)	0.033 (2)	−0.014 (2)	−0.0077 (19)	−0.0169 (18)
N3	0.042 (3)	0.027 (2)	0.023 (2)	−0.007 (2)	−0.0027 (18)	−0.0130 (18)
C10	0.049 (3)	0.044 (3)	0.031 (3)	−0.007 (3)	−0.005 (2)	−0.024 (3)
N4	0.046 (3)	0.031 (2)	0.040 (3)	−0.002 (2)	−0.009 (2)	−0.019 (2)
C9	0.041 (3)	0.047 (3)	0.037 (3)	−0.002 (3)	0.001 (2)	−0.025 (3)
C7	0.046 (3)	0.041 (3)	0.037 (3)	0.001 (3)	−0.014 (3)	−0.016 (3)
C6	0.050 (3)	0.029 (3)	0.031 (3)	0.001 (3)	−0.015 (2)	−0.016 (2)
C8	0.047 (4)	0.039 (3)	0.043 (3)	0.002 (3)	−0.009 (3)	−0.020 (3)
Ni3	0.0357 (5)	0.0258 (5)	0.0275 (5)	−0.0032 (4)	−0.0080 (4)	−0.0148 (4)
O2W	0.046 (2)	0.043 (2)	0.034 (2)	−0.0071 (19)	−0.0093 (17)	−0.0240 (18)
N5	0.058 (3)	0.045 (3)	0.039 (3)	−0.017 (3)	−0.008 (2)	−0.023 (2)
O7W	0.037 (2)	0.102 (4)	0.104 (4)	0.006 (2)	−0.008 (2)	−0.089 (3)
N6	0.050 (3)	0.050 (3)	0.056 (3)	0.008 (3)	−0.023 (2)	−0.037 (3)
C14	0.042 (4)	0.071 (5)	0.079 (5)	0.013 (3)	−0.014 (3)	−0.059 (4)
O6W	0.052 (3)	0.059 (3)	0.042 (2)	0.004 (2)	−0.0005 (19)	−0.023 (2)
O5W	0.035 (2)	0.041 (2)	0.041 (2)	−0.0007 (18)	−0.0030 (16)	−0.0199 (18)
O1	0.045 (2)	0.0221 (18)	0.0296 (19)	0.0041 (16)	−0.0121 (16)	−0.0115 (15)
O6	0.042 (2)	0.026 (2)	0.044 (2)	−0.0058 (18)	−0.0156 (17)	−0.0044 (17)
C17	0.023 (2)	0.022 (2)	0.021 (2)	0.000 (2)	0.0004 (19)	−0.010 (2)
C16	0.028 (3)	0.020 (2)	0.019 (2)	0.000 (2)	−0.0066 (19)	−0.010 (2)
C18	0.025 (3)	0.022 (2)	0.028 (3)	−0.004 (2)	−0.005 (2)	−0.014 (2)
C40	0.025 (3)	0.034 (3)	0.031 (3)	0.001 (2)	−0.007 (2)	−0.015 (2)
C1	0.048 (3)	0.030 (3)	0.043 (3)	0.010 (3)	−0.016 (3)	−0.025 (3)
C28	0.036 (3)	0.027 (3)	0.032 (3)	−0.007 (2)	0.009 (2)	−0.017 (2)
C31	0.021 (3)	0.024 (3)	0.041 (3)	0.000 (2)	0.004 (2)	−0.011 (2)
C13	0.090 (5)	0.038 (4)	0.060 (4)	0.013 (4)	−0.044 (4)	−0.020 (3)
C15	0.041 (4)	0.070 (5)	0.058 (4)	−0.014 (3)	0.006 (3)	−0.046 (4)
C11	0.087 (5)	0.057 (4)	0.042 (4)	−0.035 (4)	−0.005 (3)	−0.017 (3)
C27	0.035 (3)	0.023 (3)	0.031 (3)	0.000 (2)	0.006 (2)	−0.013 (2)
C12	0.106 (6)	0.034 (4)	0.048 (4)	−0.015 (4)	−0.034 (4)	−0.002 (3)
C4	0.039 (3)	0.035 (3)	0.034 (3)	−0.003 (3)	−0.011 (2)	−0.014 (3)

### Geometric parameters (Å, º)

**Table:**

Ni1—N1^i^	2.067 (4)	C24—H24A	0.9800
Ni1—N1	2.067 (4)	C24—H24B	0.9800
Ni1—N2	2.064 (4)	C24—H24C	0.9800
Ni1—N2^i^	2.064 (4)	Ni2—N3^ii^	2.072 (4)
Ni1—O1^i^	2.134 (3)	Ni2—N3	2.072 (4)
Ni1—O1	2.134 (3)	Ni2—N4	2.076 (4)
P2—O5	1.495 (4)	Ni2—N4^ii^	2.076 (4)
P2—C29	1.804 (5)	Ni2—O1W^ii^	2.105 (4)
P2—O4	1.502 (3)	Ni2—O1W	2.105 (4)
P2—O6	1.576 (4)	O1W—H1WA	0.8701
P1—O3	1.570 (4)	O1W—H1WB	0.8691
P1—O2	1.518 (3)	N3—H3	1.0000
P1—O1	1.483 (3)	N3—C10	1.481 (6)
P1—C16	1.811 (5)	N3—C6	1.479 (6)
P3—C38	1.813 (5)	C10—H10A	0.9900
P3—O9	1.562 (4)	C10—H10B	0.9900
P3—O7	1.506 (4)	C10—C9^ii^	1.496 (7)
P3—O8	1.499 (4)	N4—H4	1.0000
C29—C30	1.391 (7)	N4—C9	1.486 (6)
C29—C28	1.381 (7)	N4—C8	1.457 (7)
O3W—H3WA	0.8523	C9—H9A	0.9900
O3W—H3WB	0.8700	C9—H9B	0.9900
O4W—H4WA	0.8687	C7—H7A	0.9900
O4W—H4WB	0.8702	C7—H7B	0.9900
C22—C41	1.400 (6)	C7—C6	1.504 (7)
C22—C23	1.402 (6)	C7—C8	1.513 (7)
C22—C19	1.497 (6)	C6—H6A	0.9900
C38—C39	1.380 (7)	C6—H6B	0.9900
C38—C37	1.390 (7)	C8—H8A	0.9900
C26—C25	1.488 (6)	C8—H8B	0.9900
C26—C31	1.388 (6)	Ni3—N5	2.070 (4)
C26—C27	1.392 (6)	Ni3—N5^iii^	2.070 (5)
N1—H1	1.0000	Ni3—N6	2.056 (5)
N1—C5	1.482 (6)	Ni3—N6^iii^	2.056 (5)
N1—C1	1.474 (6)	Ni3—O2W^iii^	2.137 (3)
O3—H3C	0.8400	Ni3—O2W	2.136 (3)
C34—C35	1.497 (6)	O2W—H2WA	0.8638
C34—C41	1.414 (6)	O2W—H2WB	0.8553
C34—C32	1.401 (6)	N5—H5	1.0000
N2—H2	1.0000	N5—C15	1.497 (7)
N2—C3	1.469 (6)	N5—C11	1.431 (8)
N2—C4	1.477 (6)	O7W—H7WA	0.8613
C3—H3A	0.9900	O7W—H7WB	0.8521
C3—H3B	0.9900	N6—H6	1.0000
C3—C2	1.521 (7)	N6—C14	1.452 (7)
O9—H9C	0.8400	N6—C13	1.487 (8)
C35—C36	1.368 (7)	C14—H14A	0.9900
C35—C40	1.392 (6)	C14—H14B	0.9900
C41—C42	1.507 (6)	C14—C15^iii^	1.507 (9)
C23—C25	1.396 (6)	O6W—H6WA	0.8706
C23—C24	1.510 (6)	O6W—H6WB	0.8688
C30—H30	0.9500	O5W—H5WA	0.8696
C30—C31	1.382 (7)	O5W—H5WB	0.8704
C25—C32	1.409 (6)	O6—H6C	0.8400
C5—H5A	0.9900	C17—H17	0.9500
C5—H5B	0.9900	C17—C16	1.387 (6)
C5—C4^i^	1.513 (7)	C17—C18	1.396 (6)
C33—H33A	0.9800	C18—H18	0.9500
C33—H33B	0.9800	C40—H40	0.9500
C33—H33C	0.9800	C1—H1A	0.9900
C33—C32	1.506 (6)	C1—H1B	0.9900
C39—H39	0.9500	C28—H28	0.9500
C39—C40	1.399 (6)	C28—C27	1.375 (7)
C42—H42A	0.9800	C31—H31	0.9500
C42—H42B	0.9800	C13—H13A	0.9900
C42—H42C	0.9800	C13—H13B	0.9900
C36—H36	0.9500	C13—C12	1.506 (9)
C36—C37	1.385 (6)	C15—H15A	0.9900
C19—C20	1.391 (6)	C15—H15B	0.9900
C19—C18	1.393 (6)	C11—H11A	0.9900
C21—H21	0.9500	C11—H11B	0.9900
C21—C20	1.389 (6)	C11—C12	1.514 (10)
C21—C16	1.398 (6)	C27—H27	0.9500
C20—H20	0.9500	C12—H12A	0.9900
C2—H2A	0.9900	C12—H12B	0.9900
C2—H2B	0.9900	C4—H4A	0.9900
C2—C1	1.513 (7)	C4—H4B	0.9900
C37—H37	0.9500		
			
N1—Ni1—N1^i^	180.0	N4—Ni2—O1W	89.10 (17)
N1—Ni1—O1^i^	91.79 (14)	N4^ii^—Ni2—O1W	90.90 (17)
N1^i^—Ni1—O1^i^	88.21 (14)	N4^ii^—Ni2—O1W^ii^	89.10 (17)
N1^i^—Ni1—O1	91.79 (14)	N4—Ni2—N4^ii^	180.00 (13)
N1—Ni1—O1	88.21 (14)	Ni2—O1W—H1WA	106.8
N1—Ni1—N2^i^	85.31 (16)	Ni2—O1W—H1WB	108.1
N1^i^—Ni1—N2	85.31 (16)	H1WA—O1W—H1WB	104.5
N1^i^—Ni1—N2^i^	94.69 (16)	Ni2—N3—H3	107.3
N1—Ni1—N2	94.69 (16)	C10—N3—Ni2	105.7 (3)
N2^i^—Ni1—N2	180.0	C10—N3—H3	107.3
N2^i^—Ni1—O1	90.47 (15)	C6—N3—Ni2	114.6 (3)
N2—Ni1—O1	89.53 (15)	C6—N3—H3	107.3
N2^i^—Ni1—O1^i^	89.53 (15)	C6—N3—C10	114.3 (4)
N2—Ni1—O1^i^	90.47 (15)	N3—C10—H10A	109.9
O1^i^—Ni1—O1	180.0	N3—C10—H10B	109.9
O5—P2—C29	110.3 (2)	N3—C10—C9^ii^	109.1 (4)
O5—P2—O4	115.4 (2)	H10A—C10—H10B	108.3
O5—P2—O6	111.3 (2)	C9^ii^—C10—H10A	109.9
O4—P2—C29	108.0 (2)	C9^ii^—C10—H10B	109.9
O4—P2—O6	110.5 (2)	Ni2—N4—H4	106.9
O6—P2—C29	100.2 (2)	C9—N4—Ni2	106.4 (3)
O3—P1—C16	102.1 (2)	C9—N4—H4	106.9
O2—P1—O3	110.2 (2)	C8—N4—Ni2	115.2 (3)
O2—P1—C16	107.1 (2)	C8—N4—H4	106.9
O1—P1—O3	111.4 (2)	C8—N4—C9	114.0 (4)
O1—P1—O2	115.2 (2)	C10^ii^—C9—H9A	110.1
O1—P1—C16	110.06 (19)	C10^ii^—C9—H9B	110.1
O9—P3—C38	101.5 (2)	N4—C9—C10^ii^	108.1 (4)
O7—P3—C38	107.6 (2)	N4—C9—H9A	110.1
O7—P3—O9	109.8 (2)	N4—C9—H9B	110.1
O8—P3—C38	109.4 (2)	H9A—C9—H9B	108.4
O8—P3—O9	111.8 (2)	H7A—C7—H7B	107.3
O8—P3—O7	115.7 (2)	C6—C7—H7A	108.1
C30—C29—P2	120.9 (4)	C6—C7—H7B	108.1
C28—C29—P2	121.0 (4)	C6—C7—C8	116.9 (5)
C28—C29—C30	118.0 (4)	C8—C7—H7A	108.1
H3WA—O3W—H3WB	98.3	C8—C7—H7B	108.1
H4WA—O4W—H4WB	104.4	N3—C6—C7	112.6 (4)
C41—C22—C23	120.1 (4)	N3—C6—H6A	109.1
C41—C22—C19	118.6 (4)	N3—C6—H6B	109.1
C23—C22—C19	120.9 (4)	C7—C6—H6A	109.1
C39—C38—P3	121.3 (4)	C7—C6—H6B	109.1
C39—C38—C37	118.6 (4)	H6A—C6—H6B	107.8
C37—C38—P3	120.1 (4)	N4—C8—C7	111.9 (5)
C31—C26—C25	123.6 (4)	N4—C8—H8A	109.2
C31—C26—C27	116.3 (4)	N4—C8—H8B	109.2
C27—C26—C25	120.1 (4)	C7—C8—H8A	109.2
Ni1—N1—H1	106.9	C7—C8—H8B	109.2
C5—N1—Ni1	104.8 (3)	H8A—C8—H8B	107.9
C5—N1—H1	106.9	O2W—Ni3—O2W^iii^	180.0
C1—N1—Ni1	116.4 (3)	N5^iii^—Ni3—O2W	91.54 (15)
C1—N1—H1	106.9	N5—Ni3—O2W	88.46 (15)
C1—N1—C5	114.2 (4)	N5^iii^—Ni3—O2W^iii^	88.46 (15)
P1—O3—H3C	109.5	N5—Ni3—O2W^iii^	91.54 (15)
C41—C34—C35	117.8 (4)	N5—Ni3—N5^iii^	180.0
C32—C34—C35	121.4 (4)	N6—Ni3—O2W	89.19 (16)
C32—C34—C41	120.8 (4)	N6^iii^—Ni3—O2W	90.81 (16)
Ni1—N2—H2	106.6	N6^iii^—Ni3—O2W^iii^	89.19 (16)
C3—N2—Ni1	116.1 (3)	N6—Ni3—O2W^iii^	90.81 (16)
C3—N2—H2	106.6	N5—Ni3—N6^iii^	85.2 (2)
C3—N2—C4	114.4 (4)	N5—Ni3—N6	94.8 (2)
C4—N2—Ni1	106.0 (3)	N5^iii^—Ni3—N6	85.2 (2)
C4—N2—H2	106.6	N5^iii^—Ni3—N6^iii^	94.8 (2)
N2—C3—H3A	109.1	N6^iii^—Ni3—N6	180.0
N2—C3—H3B	109.1	Ni3—O2W—H2WA	110.2
N2—C3—C2	112.3 (4)	Ni3—O2W—H2WB	109.7
H3A—C3—H3B	107.9	H2WA—O2W—H2WB	103.1
C2—C3—H3A	109.1	Ni3—N5—H5	106.1
C2—C3—H3B	109.1	C15—N5—Ni3	105.8 (4)
P3—O9—H9C	109.5	C15—N5—H5	106.1
C36—C35—C34	119.7 (4)	C11—N5—Ni3	117.4 (4)
C36—C35—C40	118.5 (4)	C11—N5—H5	106.1
C40—C35—C34	121.7 (4)	C11—N5—C15	114.5 (5)
C22—C41—C34	119.4 (4)	H7WA—O7W—H7WB	94.0
C22—C41—C42	119.5 (4)	Ni3—N6—H6	106.1
C34—C41—C42	121.1 (4)	C14—N6—Ni3	107.9 (4)
C22—C23—C24	118.7 (4)	C14—N6—H6	106.1
C25—C23—C22	120.0 (4)	C14—N6—C13	114.0 (5)
C25—C23—C24	121.2 (4)	C13—N6—Ni3	115.9 (4)
C29—C30—H30	119.4	C13—N6—H6	106.1
C31—C30—C29	121.2 (5)	N6—C14—H14A	109.8
C31—C30—H30	119.4	N6—C14—H14B	109.8
C23—C25—C26	118.9 (4)	N6—C14—C15^iii^	109.4 (5)
C23—C25—C32	120.8 (4)	H14A—C14—H14B	108.2
C32—C25—C26	120.2 (4)	C15^iii^—C14—H14A	109.8
N1—C5—H5A	110.0	C15^iii^—C14—H14B	109.8
N1—C5—H5B	110.0	H6WA—O6W—H6WB	104.5
N1—C5—C4^i^	108.4 (4)	H5WA—O5W—H5WB	104.5
H5A—C5—H5B	108.4	P1—O1—Ni1	167.3 (2)
C4^i^—C5—H5A	110.0	P2—O6—H6C	109.5
C4^i^—C5—H5B	110.0	C16—C17—H17	119.7
H33A—C33—H33B	109.5	C16—C17—C18	120.5 (4)
H33A—C33—H33C	109.5	C18—C17—H17	119.7
H33B—C33—H33C	109.5	C21—C16—P1	122.4 (4)
C32—C33—H33A	109.5	C17—C16—P1	118.7 (3)
C32—C33—H33B	109.5	C17—C16—C21	118.7 (4)
C32—C33—H33C	109.5	C19—C18—C17	121.1 (4)
C38—C39—H39	119.6	C19—C18—H18	119.5
C38—C39—C40	120.8 (5)	C17—C18—H18	119.5
C40—C39—H39	119.6	C35—C40—C39	120.0 (5)
C41—C42—H42A	109.5	C35—C40—H40	120.0
C41—C42—H42B	109.5	C39—C40—H40	120.0
C41—C42—H42C	109.5	N1—C1—C2	112.0 (4)
H42A—C42—H42B	109.5	N1—C1—H1A	109.2
H42A—C42—H42C	109.5	N1—C1—H1B	109.2
H42B—C42—H42C	109.5	C2—C1—H1A	109.2
C35—C36—H36	119.1	C2—C1—H1B	109.2
C35—C36—C37	121.8 (5)	H1A—C1—H1B	107.9
C37—C36—H36	119.1	C29—C28—H28	120.0
C20—C19—C22	124.0 (4)	C27—C28—C29	120.1 (5)
C20—C19—C18	117.9 (4)	C27—C28—H28	120.0
C18—C19—C22	118.0 (4)	C26—C31—H31	119.3
C20—C21—H21	119.9	C30—C31—C26	121.4 (4)
C20—C21—C16	120.2 (4)	C30—C31—H31	119.3
C16—C21—H21	119.9	N6—C13—H13A	109.2
C19—C20—H20	119.3	N6—C13—H13B	109.2
C21—C20—C19	121.5 (4)	N6—C13—C12	112.1 (5)
C21—C20—H20	119.3	H13A—C13—H13B	107.9
C3—C2—H2A	108.4	C12—C13—H13A	109.2
C3—C2—H2B	108.4	C12—C13—H13B	109.2
H2A—C2—H2B	107.5	N5—C15—C14^iii^	110.1 (5)
C1—C2—C3	115.4 (4)	N5—C15—H15A	109.6
C1—C2—H2A	108.4	N5—C15—H15B	109.6
C1—C2—H2B	108.4	C14^iii^—C15—H15A	109.6
C38—C37—H37	120.0	C14^iii^—C15—H15B	109.6
C36—C37—C38	120.1 (5)	H15A—C15—H15B	108.2
C36—C37—H37	120.0	N5—C11—H11A	109.3
C34—C32—C25	118.8 (4)	N5—C11—H11B	109.3
C34—C32—C33	120.2 (4)	N5—C11—C12	111.5 (5)
C25—C32—C33	121.0 (4)	H11A—C11—H11B	108.0
C23—C24—H24A	109.5	C12—C11—H11A	109.3
C23—C24—H24B	109.5	C12—C11—H11B	109.3
C23—C24—H24C	109.5	C26—C27—H27	118.5
H24A—C24—H24B	109.5	C28—C27—C26	123.0 (5)
H24A—C24—H24C	109.5	C28—C27—H27	118.5
H24B—C24—H24C	109.5	C13—C12—C11	118.5 (6)
O1W^ii^—Ni2—O1W	180.0	C13—C12—H12A	107.7
N3—Ni2—O1W	88.65 (15)	C13—C12—H12B	107.7
N3^ii^—Ni2—O1W^ii^	88.65 (15)	C11—C12—H12A	107.7
N3—Ni2—O1W^ii^	91.35 (15)	C11—C12—H12B	107.7
N3^ii^—Ni2—O1W	91.35 (15)	H12A—C12—H12B	107.1
N3—Ni2—N3^ii^	180.0	N2—C4—C5^i^	107.4 (4)
N3—Ni2—N4^ii^	84.66 (16)	N2—C4—H4A	110.2
N3^ii^—Ni2—N4^ii^	95.34 (16)	N2—C4—H4B	110.2
N3^ii^—Ni2—N4	84.66 (16)	C5^i^—C4—H4A	110.2
N3—Ni2—N4	95.34 (16)	C5^i^—C4—H4B	110.2
N4—Ni2—O1W^ii^	90.90 (17)	H4A—C4—H4B	108.5
			
Ni1—N1—C5—C4^i^	44.0 (4)	O7—P3—C38—C37	38.0 (5)
Ni1—N1—C1—C2	−55.0 (5)	C36—C35—C40—C39	−2.7 (7)
Ni1—N2—C3—C2	55.3 (5)	C19—C22—C41—C34	169.3 (4)
Ni1—N2—C4—C5^i^	−42.6 (4)	C19—C22—C41—C42	−9.3 (6)
P2—C29—C30—C31	−177.8 (4)	C19—C22—C23—C25	−169.7 (4)
P2—C29—C28—C27	177.0 (4)	C19—C22—C23—C24	12.3 (6)
P3—C38—C39—C40	−176.5 (4)	O8—P3—C38—C39	−15.9 (5)
P3—C38—C37—C36	177.2 (4)	O8—P3—C38—C37	164.4 (4)
O5—P2—C29—C30	29.0 (5)	C20—C19—C18—C17	−1.5 (7)
O5—P2—C29—C28	−149.2 (4)	C20—C21—C16—P1	173.3 (4)
C29—C30—C31—C26	0.7 (8)	C20—C21—C16—C17	−2.0 (7)
C29—C28—C27—C26	1.1 (8)	C37—C38—C39—C40	3.2 (7)
O4—P2—C29—C30	−98.0 (4)	C32—C34—C35—C36	−105.9 (5)
O4—P2—C29—C28	83.8 (4)	C32—C34—C35—C40	77.4 (6)
C22—C23—C25—C26	175.0 (4)	C32—C34—C41—C22	2.2 (7)
C22—C23—C25—C32	−1.0 (7)	C32—C34—C41—C42	−179.2 (4)
C22—C19—C20—C21	−174.9 (4)	C24—C23—C25—C26	−7.1 (7)
C22—C19—C18—C17	175.5 (4)	C24—C23—C25—C32	176.9 (4)
C38—C39—C40—C35	−0.6 (8)	Ni2—N3—C10—C9^ii^	43.0 (5)
C26—C25—C32—C34	−177.1 (4)	Ni2—N3—C6—C7	−54.7 (5)
C26—C25—C32—C33	2.1 (7)	Ni2—N4—C9—C10^ii^	−41.1 (5)
O3—P1—O1—Ni1	149.1 (9)	Ni2—N4—C8—C7	55.8 (5)
O3—P1—C16—C21	25.1 (4)	C10—N3—C6—C7	−176.9 (4)
O3—P1—C16—C17	−159.6 (3)	C9—N4—C8—C7	179.2 (5)
C34—C35—C36—C37	−173.4 (4)	C6—N3—C10—C9^ii^	170.0 (4)
C34—C35—C40—C39	174.0 (4)	C6—C7—C8—N4	−71.7 (6)
N2—C3—C2—C1	−71.6 (6)	C8—N4—C9—C10^ii^	−169.2 (4)
C3—N2—C4—C5^i^	−171.9 (4)	C8—C7—C6—N3	71.3 (6)
C3—C2—C1—N1	71.1 (6)	Ni3—N5—C15—C14^iii^	37.7 (5)
O2—P1—O1—Ni1	22.6 (10)	Ni3—N5—C11—C12	−53.9 (6)
O2—P1—C16—C21	141.0 (4)	Ni3—N6—C14—C15^iii^	−39.2 (5)
O2—P1—C16—C17	−43.8 (4)	Ni3—N6—C13—C12	52.9 (6)
O9—P3—C38—C39	102.4 (4)	N5—C11—C12—C13	69.6 (7)
O9—P3—C38—C37	−77.3 (4)	N6—C13—C12—C11	−69.2 (7)
C35—C34—C41—C22	−175.0 (4)	C14—N6—C13—C12	178.9 (5)
C35—C34—C41—C42	3.6 (7)	O1—P1—C16—C21	−93.2 (4)
C35—C34—C32—C25	177.6 (4)	O1—P1—C16—C17	82.0 (4)
C35—C34—C32—C33	−1.6 (7)	O6—P2—C29—C30	146.4 (4)
C35—C36—C37—C38	−0.8 (8)	O6—P2—C29—C28	−31.8 (4)
C41—C22—C23—C25	3.8 (7)	C16—P1—O1—Ni1	−98.5 (10)
C41—C22—C23—C24	−174.2 (4)	C16—C21—C20—C19	−0.2 (7)
C41—C22—C19—C20	85.7 (6)	C16—C17—C18—C19	−0.7 (7)
C41—C22—C19—C18	−91.1 (5)	C18—C19—C20—C21	1.9 (7)
C41—C34—C35—C36	71.2 (6)	C18—C17—C16—P1	−173.0 (3)
C41—C34—C35—C40	−105.5 (5)	C18—C17—C16—C21	2.4 (7)
C41—C34—C32—C25	0.6 (7)	C40—C35—C36—C37	3.4 (7)
C41—C34—C32—C33	−178.6 (4)	C1—N1—C5—C4^i^	172.6 (4)
C23—C22—C41—C34	−4.4 (7)	C28—C29—C30—C31	0.4 (8)
C23—C22—C41—C42	177.0 (4)	C31—C26—C25—C23	110.8 (5)
C23—C22—C19—C20	−100.7 (5)	C31—C26—C25—C32	−73.2 (6)
C23—C22—C19—C18	82.5 (5)	C31—C26—C27—C28	0.1 (7)
C23—C25—C32—C34	−1.2 (7)	C13—N6—C14—C15^iii^	−169.4 (5)
C23—C25—C32—C33	178.0 (4)	C15—N5—C11—C12	−179.0 (5)
C30—C29—C28—C27	−1.3 (7)	C11—N5—C15—C14^iii^	168.7 (5)
C25—C26—C31—C30	178.9 (5)	C27—C26—C25—C23	−69.4 (6)
C25—C26—C27—C28	−179.7 (5)	C27—C26—C25—C32	106.6 (5)
C5—N1—C1—C2	−177.5 (4)	C27—C26—C31—C30	−0.9 (7)
C39—C38—C37—C36	−2.5 (7)	C4—N2—C3—C2	179.3 (5)
O7—P3—C38—C39	−142.3 (4)		

Symmetry codes: (i) −*x*+2, −*y*+1, −*z*+2; (ii) −*x*+2, −*y*+2, −*z*+1; (iii) −*x*+1, −*y*+3, −*z*+1.

### Hydrogen-bond geometry (Å, º)

**Table:**

*D*—H···*A*	*D*—H	H···*A*	*D*···*A*	*D*—H···*A*
N1—H1···O6^iv^	1.00	2.32	3.196 (5)	146
N2—H2···O6*W*	1.00	2.18	3.039 (6)	143
N3—H3···07^v^	1.00	2.13	3.102 (6)	162
N4—H4···O4*W*	1.00	2.06	3.056 (6)	173
N5—H5···O9^vi^	1.00	2.07	3.003 (6)	155
N6—H6···O7*W*^ii^	1.00	1.98	2.956 (6)	166
O3—H3*C*···O5^iv^	0.84	1.84	2.654 (5)	162
O6—H6*C*···O3*W*^vii^	0.84	1.75	2.550 (5)	159
O9—H9*C*···O4^viii^	0.84	1.74	2.517 (5)	154
O1*W*—H1*WB*···O7^v^	0.87	1.81	2.679 (5)	173
O1*W*—H1*WA*···O4*W*	0.87	2.45	3.256 (6)	155
O2*W*—H2*WB*···O4	0.86	1.90	2.729 (5)	164
O2*W*—H2*WA*···O7*W*^ix^	0.86	1.81	2.675 (6)	174
O3*W*—H3*WB*···O2	0.87	1.81	2.676 (4)	177
O3*W*—H3*WA*···O7^v^	0.85	1.84	2.689 (5)	174
O4*W*—H4*WB*···O3	0.87	2.26	3.115 (6)	167
O4*W*—H4*WA*···O8^v^	0.87	1.93	2.796 (6)	172
O5*W*—H5*WB*···O5^x^	0.87	1.98	2.813 (5)	159
O5*W*—H5*WA*···O8^xi^	0.87	1.87	2.725 (5)	168
O6*W*—H6*WB*···O2	0.87	2.02	2.799 (6)	149
O6*W*—H6*WA*···O5*W*	0.87	2.00	2.842 (5)	164
O7*W*—H7*WB*···O3*W*	0.85	2.02	2.731 (5)	140
O7*W*—H7*WA*···O5*W*	0.86	1.83	2.688 (5)	173

Symmetry codes: (ii) −*x*+2, −*y*+2, −*z*+1; (iv) *x*, *y*−1, *z*; (v) *x*, *y*, *z*−1; (vi) −*x*+1, −*y*+2, −*z*+2; (vii) *x*, *y*+1, *z*; (viii) *x*, *y*−1, *z*+1; (ix) *x*−1, *y*+1, *z*; (x) *x*+1, *y*−1, *z*; (xi) *x*+1, *y*, *z*−1.
